# Spectroscopic Evidence of the Improvement of Reactive Iron Mineral Content in Red Soil by Long-Term Application of Swine Manure

**DOI:** 10.1371/journal.pone.0146364

**Published:** 2016-01-11

**Authors:** Chichao Huang, Sha Liu, Ruizhi Li, Fusheng Sun, Ying Zhou, Guanghui Yu

**Affiliations:** 1 National Engineering Research Center for Organic-based Fertilizers, Jiangsu Collaborative Innovation Center for Solid Organic Waste Resource Utilization, Jiangsu Provincial Key Lab for Organic Solid Waste Utilization, Nanjing Agricultural University, Nanjing 210095, PR China; 2 Shanghai Institute of Measurement and Testing Technology, Shanghai 201203, China; Southwest University, CHINA

## Abstract

Mineral elements in soil solutions are thought to be the precursor of the formation of reactive minerals, which play an important role in global carbon (C) cycling. However, information regarding the regulation of mineral elements release in soil is scarce. Here, we examined the long-term (i.e., 23 yrs) effects of fertilisation practices on Fe minerals in a red soil in Southern China. The results from chemical analysis and Fourier-transform infrared spectroscopy showed that long-term swine manure (M) treatment released greater amounts of minerals into soil solutions than chemical fertilisers (NPK) treatment, and Fe played a dominant role in the preservation of dissolved organic C. Furthermore, Fe K-edge X-ray absorption near-edge fine structure spectroscopy demonstrated that reactive Fe minerals were mainly composed of less crystalline ferrihydrite in the M-treated soil and more crystalline goethite in the NPK-treated soil. In conclusion, this study reported spectroscopic evidence of the improvement of reactive Femineral content in the M-treated soil colloids when compared to NPK-treated soil colloids.

## Introduction

Globally, soil organic matter (SOM) contained greater than three-fold more carbon (C) than the atmosphere and terrestrial vegetation [1]. The biogeochemical cycles of organic C and iron (Fe) were strongly interlinked [2,3]. Lalonde et al. [2] suggested that approximately 21.5% of the organic carbon in soil or sediment was directly bound to reactive Fe minerals. Therefore, reactive Fe minerals could play an important role in the long-term storage of organic C and thus the dynamics of the global C cycle. However, information concerning the mechanisms that regulate reactive Fe minerals in soils is scarce.

Recently, a study of two-century land use changes on soil iron crystallinity and accumulation [4] suggested that land use changes (agriculture and reforestation) play an important role in transforming the iron crystallinity and its interaction with organic matter decomposition in soils. By evaluating soil C accumulation for 3 years across a 7-year chronosequence of three farms converted to management-intensive grazing, Machmuller et al. [5] showed that soil cation exchange capacity linearly increased with soil carbon accumulation within a decade of management-intensive grazing practices. This result suggested mineral elements (i.e., soil cations) could be affected by agricultural practices. Furthermore, Keiluweit et al. [6] indicated that root exudates might affect the formation of reactive minerals. Also, our recent investigations demonstrated that long-term organic fertilisation treatments could increase the concentrations of reactive minerals (i.e., non-crystalline Fe [7] and allophane [8,9]) in red soils in Southern China. However, non-crystalline Fe contributed only a portion of reactive Fe minerals present in the soil, which were usually defined as Fe extracted with oxalate or citrate-bicarbonate-dithionite (CBD) and might not serve to characterise the total amount of reactive Fe minerals. A better understanding of the effect of fertilisation practices on reactive iron oxides is important for predicting and managing C preservation in soils.

X-ray absorption near-edge fine structure (XANES) spectroscopy is an element specific technique that is sensitive to the oxidation state and to the local structure of the absorber element [10]. Using hard X-rays at the Fe K-edge, this technique provided a powerful tool to not only identify but also quantify the various Fe phases present in soils, which could be very complex and might mask magnetically weak phases when examined by Mössbauer spectroscopy or X-ray magnetic circular dichroism [11]. However, the composition of reactive Fe minerals in long-term fertilised soils remains poorly understood.

The objectives of this study were 1) to examine the effect of long-term fertlisation regimes on the composition of reactive Fe minerals in these soils, 2) to test which functional groups in soil C were preferentially binding with reactive iron oxides, and 3) to mimic Fe mineral transformation with the addition of organic acids. For these purposes, four contrasting fertilisation regimes that each supplied 300 kg N/ha/year (two crops, wheat and corn) were examined during a long-term (i.e., 23 yrs) fertilisation experiment: i) no fertiliser (Control), ii) inorganic chemical fertilisers of nitrogen, phosphorus and potassium only (NPK or chemical fertilisation hereafter), iii) swine manure only (M), and iv) a combination of swine manure and NPK fertilisers (MNPK) (M and MNPK are collectively called organic fertilisation hereafter).

## Materials and Methods

### 2.1 Sample source and handling

Soil samples were collected from a depth of 0–20 cm in September 2013 from the Qiyang Experiment using a 5-cm internal diameter auger. The long-term fertilisation experiment was established on a Ferralic Cambisol soil in September 1990 at the Qiyang Experimental Station of the Chinese Academy of Agricultural Sciences, Hunan, China (26°45’N and 111°52’E, 120 m above sea level) [8, 12]. The mean values for annual temperature, evaporation, frost-free days and sunshine hours were 18°C, 1,470 mm, 300 d and 1,610 h, respectively, and the site received 1,255 mm of mean annual precipitation, approximately 70%–80% of which falls from April to October. No specific field permits were required for this study. The land accessed is not privately owned or protected. No protected species were sampled. A detailed description of the long-term fertilisation experiment site has been provided previously [8,13]. According to the Food and Agriculture Organization of the United Nations (FAO) classification, the red soil is a Ferralic Cambisol [14]. The top soil contains approximately 61.4% clay, 34.9% silt, and 3.7% sand. Each plot was 20 m long and 10 m wide with a 1.0-m deep cement barrier zone between each plot. Each plot was separated into three equal-sized regions, and 10 cores were randomly sampled from each region. We carefully selected four fertilisation treatments for this study: Control, NPK, NPKM, and M. The N fertilizer was provided as urea at 300 kg N ha^−1^, P as single superphosphate at 53 kg P ha^−1^, and K as KCl at 100 kg K ha^−1^ before crop planting. The N content of the manure was 16.7 g kg^−1^ in dry weight. The ratio of organic N in manure compared to that added as inorganic N in fertilizer was 2.3:1. Thirty percent of the total amount of individual fertilizer applied each year was used for wheat and 70% for corn. The experimental plots were completely dependent on precipitation.

Fresh soil was thoroughly mixed, air-dried, and sieved through a 5-mm screen for further analysis. The air-dried samples were passed through a 2-mm screen prior to sample storage, passed through a 0.25-mm screen before pH, SOC, and C/N measurements, and passed through a 0.15-mm screen before extraction experiments.

Soil colloids were isolated using the following procedure [15]. Briefly, air-dried soil was suspended in deionised water at the ratio of 1:5 (w/v), shaken for 8 h at 25°C, and centrifuged for 6 min at 2,500 *g*. Aliquots of the supernatant suspensions containing the soil colloids were transferred into 50-mL glass vials, stored in the dark at 4°C, and analysed within a few days.

### 2.2 Chemical analysis

DOC was measured using a TOC/TN analyser (multi N/C 3000, Analytik Jena AG, Germany). The main metal ions in the colloids were quantified after digestion. The following procedure was performed: first, the soil colloids were mixed with 10% nitric acid at a ratio of 1:1 (v/v) on a heating plate [16]; then, the mixture was heated to 150°C and held there for 2 hrs. After digestion, the mixture was filtered through a filtration membrane (0.45 μm) and stored in a suitable container. The main mineral elements, namely, Fe, Al, Ca, Mg and Si, were quantified by inductively coupled plasma atomic emission spectroscopy (ICP-AES).

The extraction of reactive Fe minerals (Fe_d_) was conducted using a 0.1 M citrate-bicarbonate-dithionite (CBD) solution [2]. To determine the total Fe (Fe_t_) content in the soils, 0.5 g of powdered soil was weighed into Teflon tubes, mixed with trace metal grade acids (5 ml HNO_3_ and 10 ml HF), and carefully boiled for 1 hr at 120 ± 3°C. After cooling, 5 ml of HClO_4_ was added, and the sample was heated to 220 ± 3°C until the sample boiled to dryness; then, 5 ml of HF and 2 ml of HClO_4_ were added into the Teflon tubes. The sample was digested completely until the residue turned white and grey. Finally, the digest was dissolved with 3 ml of HNO_3_ (v/v = 1:1) and increased to a volume of 50 ml in a polypropylene centrifuge tube with deionised water [17,18]. The concentration of Fe_t_ was analysed by ICP-AES.

### 2.3 Fourier-transform infrared (FTIR) spectroscopy analysis

The soil samples and the CBD extracts were collected and freeze-dried for FTIR analysis. FTIR spectra were measured for 1 mg of freeze-dried sample added to 100 mg of potassium bromide (KBr, IR grade) and collected using a Nicolet iS10 FTIR spectrometer (Thermo Nicolet, USA) at 4 cm^-1^ of resolution after 200 scans over the range from 4,000–400 cm^-1^.

### 2.4 C 1s NEXAFS spectroscopy

The colloidal particles from the soils collected from the four fertilisation treatments were characterised by C 1s NEXAFS spectroscopy on BL08U at the Shanghai Synchrotron Radiation Facility, Shanghai Institute of Applied Physics, Chinese Academy of Sciences. For specimen preparation, one droplet of soil colloid suspension was deposited at a 100-nm thickness onto a Si_3_N_4_ window, which was previously glued onto the detection plate of the microscope. The sample thickness is important to obtain a good signal-to-noise ratio when using NEXAFS spectroscopy [19]. The main 1s-p and Rydberg/mixed valence transitions in the fine structure regions of the C K-edge spectra were recorded in the energy range from 284–310 eV. Background measurements were collected by measuring an empty Au wafer on each sample plate loaded into the chamber. The normalisation current was also measured during each scan by collecting the TEY from an Au mesh. The mesh was monitored for C contamination and was periodically refreshed using an in situ Au evaporator incorporated into the beamline vacuum system [20]. All the data were normalised prior to curve fitting using the ATHENA software (version 2.1.1) [21]. Peak resonances with specific bonding environments were assigned on the basis of the spectral signatures of pure chemical standards representative of specific functional groups [19]. The details of deconvolution have been described elsewhere [15,20,22,23]. Spectral regions represented with Gaussian curves were described as being generally attributed to the functional groups from G1 to G8, details are given in data in S1 Table.

### 2.5 Fe K-edge XAFS analysis

Fe K-edge absorption spectra were collected using an Si (111) double crystal monochromator at the XAFS station of the BL14W1 beamline of the Shanghai Synchrotron Radiation Facility (SSRF). The storage ring was operated at 3.5 GeV with an electron current that decreased from 210 to 150 mA within about 8 hrs. Samples were ground into a fine powder and brushed onto tape that was stacked together to approximately one X-ray-absorption length at the corresponding metal edges. The intensities of the incident and the transmitted X-rays were monitored in ionisation chambers filled with nitrogen gas. All of the spectra presented were measured at room temperature. Standard samples of ferrihydrite, goethite, lepidocrocite, maghemite, Fe(III) sulphate, Fe(II) sulphate, Fe(III) oxalate, and Fe(II) oxalate were recorded in the transmission mode (S1 Fig), while the prepared samples were measured in the fluorescence mode. The X-ray energy scale was calibrated to the iron K-edge (7112.0 eV) using an iron metal foil before XANES measurements were performed. The incident X-ray energy was varied from 7090 to 7180 eV in 0.5-eV increments using a monochromator for a 10 s dwell, and a 19 element high-purity Ge detector was used to collect an energy scan near the iron K edge of a given iron-containing particle. The XAFS data were processed and analysed using the ATHENA software (version 2.1.1).

### 2.6 Simulation studies on the formation of SRO Fe minerals with the addition of oxalic acid

The soil colloids solutions for the M treatment and oxalic acid solution were prepared. Oxalic acid solutions were added to the soil colloid solutions with stirring. The final concentrations of oxalic acid in the soil colloid suspensions were 10 and 100 mg/L, and the pH values were adjusted to 6.7 which were same as that of the raw soil colloids solution. After one day incubation, the suspensions in the series of reaction solutions were lyophilized under -50°C for 2 day and then used for Fe K-edge XANES measurement.

### 2.7 Statistical analysis

One-way analysis of variance (ANOVA) was employed to test the effects of long-term fertilisation on soil reactive Fe minerals. Significance was determined by performing one-way ANOVA’s followed by Tukey’s HSD post hoc tests. Conditions of normality and homogeneity of variance were met. Means ± SE (*n* = 3) followed by different letters indicate significant differences between treatments at *P* < 0.01. Values of Pearson’s correlation coefficient (*R*) were used to evaluate the linear correlations among reactive Fe minerals and C functional groups. The Pearson’s coefficient is always a number between –1 and +1, where –1 denotes a perfect negative correlation, +1 denotes a perfect positive correlation, and 0 denotes the absence of a relationship. The correlations were considered to be statistically significant at a 95% confidence interval (*P* < 0.05).

## Results

### 3.1 Characteristics of soils in contrasting fertilisation treatments

After 23 yrs of long-term fertilisation, a higher SOC (soil organic carbon) concentration was measured from the NPKM and M treatments (i.e., 12.37 ± 0.08 and 14.76 ± 0.04 g/kg, respectively) than the NPK and Control treatments (i.e., 10.62 ± 0.03 and 8.05 ± 0.05 g/kg, respectively) (Table 1). Meanwhile, the soil pH values for the NPKM and M treatments were greater than those for the NPK treatment, indicating that organic fertilisers could enhance the buffering capacity of soils while chemical fertilisers accelerate soil acidification. Compared to raw soil (i.e., the unfertilized soil prior to long-term fertilisation treatments), both organic and chemical fertilisations increased soil C pools. However, organic fertilisation improved soil pH while chemical fertilisation decreased soil pH. These SOC and pH results were similar to those reported in our previous publications [7–9], supporting the finding that organic fertilisation could increase soil C pools and enhance soil buffering capacity, while chemical fertilisation decreases soil C pools and accelerates soil acidification.

**Table 1:** Characteristics of soils from the various long-term fertilisation treatments^a^.

Treatments	SOC (g/kg)	Bulk soil pH (H_2_O)	Fe_t_ (g/kg)	Fe_d_ (mg/g)	Fe_d_/Fe_t_	DOC (mg/L)
Raw soil	8.54 ± 0.03	5.7± 0.09	53.60 ± 0.68	41.36 ± 0.74	0.77 ± 0.06	34.52 ± 1.61
Control	8.05 ± 0.05	5.47 ± 0.07	50.70 ± 1.32	40.17 ± 0.78	0.79 ± 0.03	26.17 ± 13.32
NPK	10.62 ± 0.03	4.15 ± 0.02	55.40 ± 1.89	45.18 ± 3.36	0.82 ± 0.03	22.19 ± 1.43
NPKM	12.37 ± 0.08	5.84 ± 0.02	49.31 ± 0.59	41.52 ± 5.31	0.84 ± 0.11	217.08 ± 82.95
M	14.76 ± 0.04	6.63 ± 0.05	46.72 ± 1.94	39.84 ± 1.86	0.85 ± 0.01	101.39 ± 14.15

^*a*^Control, no fertilisation;

NPK, chemical fertilisation; NPKM, chemical plus swine manure fertilisation; M, swine manure fertilisation. SOC, soil organic carbon. DOC, dissolved organic carbon. Fe_d_, the CBD extracted reactive Fe. Fe_t_, the total Fe. Fe_d_/Fe_t_, this ratio represents the iron freeness index.

**Fig 1:**
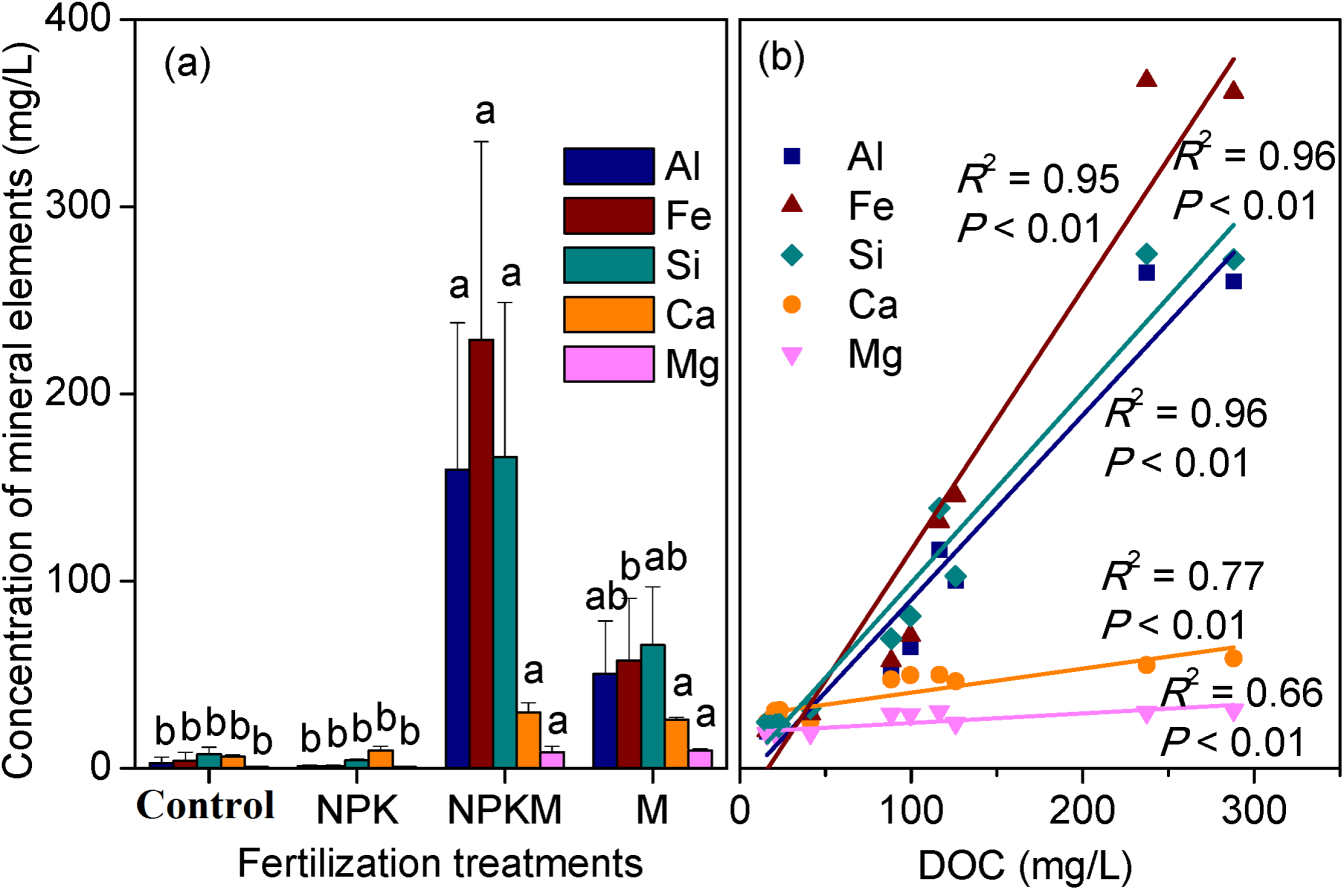
Concentrations of mineral elements in soil colloids under contrasting fertilisation treatments (a) and their correlations with dissolved organic carbon (DOC) (b). Control, no fertilisation; NPK, chemical fertilisation; NPKM, chemical plus swine manure fertilisation; M, swine manure fertilisation. Significant differences among fertilisation treatments were determined using one-way ANOVA followed by Tukey’s HSD post hoc test at *P* < 0.01; conditions of normality and homogeneity of variance were met. The slopes of the relationships between mineral elements and DOC shown in (b) clearly demonstrate that DOC is dominated by Fe, Si, and Al rather than by Ca and Mg in red soils in Southern China.

Additionally, total iron (Fe_t_) in soils exhibited an opposite trend to SOC concentration (Table 1), suggesting that total iron was not responsible for the preservation of SOC. To determine whether soluble reactive Fe played an important role in the preservation of SOC, the concentrations of mineral elements (i.e., Fe, Al, Si, Ca, and Mg) and dissolved organic C (DOC) in soil colloids were measured (Fig 1-a). Compared to raw soil, organic fertilisation increased soil DOC pool while control and chemical fertilisations decreased it. Intriguingly, the highest concentrations of both mineral elements and DOC were found in the NPKM treatment, followed by the M treatment, with the lowest concentrations observed in the Control and NPK treatments. Moreover, there are strong correlations among the mineral elements and DOC (Fig 1-b), demonstrating that all of the tested mineral elements together controlled the preservation of DOC. Importantly, the slopes of the relationships between mineral elements and DOC follow the order: Fe (1.17) > Si (0.85) > Al (0.83) >> Ca (0.11) > Mg (0.04), revealing that among the tested mineral elements, the per unit Fe was bound with more DOC than other mineral elements. Therefore, Fe played an important role in the preservation of DOC in red soils in Southern China. The higher concentrations of DOC and mineral elements in the NPKM treatment in relation to the NPK or M treatment may be due to the better growth of plants in the NPKM treatment. This would result in increased root exudate production in the NPKM treatment, which simulated higher concentration of DOC and mineral element production.

To quantify the concentration of reactive Fe, the CBD method was used to extract Fe (i.e., Fe_d_) from soils. The results demonstrated that the Fe_d_ in soils exhibited a distinct relationship with SOC (Table 1). However, the iron freeness index, indicated by the Fe_d_/Fe_t_ ratio, had a similar relationship to SOC, revealing that Fe_d_/Fe_t_ rather than Fe_d_ may be the critical factor in the determination of SOC preservation. An increase in the Fe_d_/Fe_t_ ratio suggests a high degree of soil weathering [24]. Compared to raw soil, all of the fertilisations increased the Fe_d_/Fe_t_ ratio. Therefore, organic fertilisation increased the rate of soil weathering much more than control and chemical fertilisations.

### 3.2 FTIR spectroscopy of reactive minerals under contrasting fertilisation treatments

**Fig 2:**
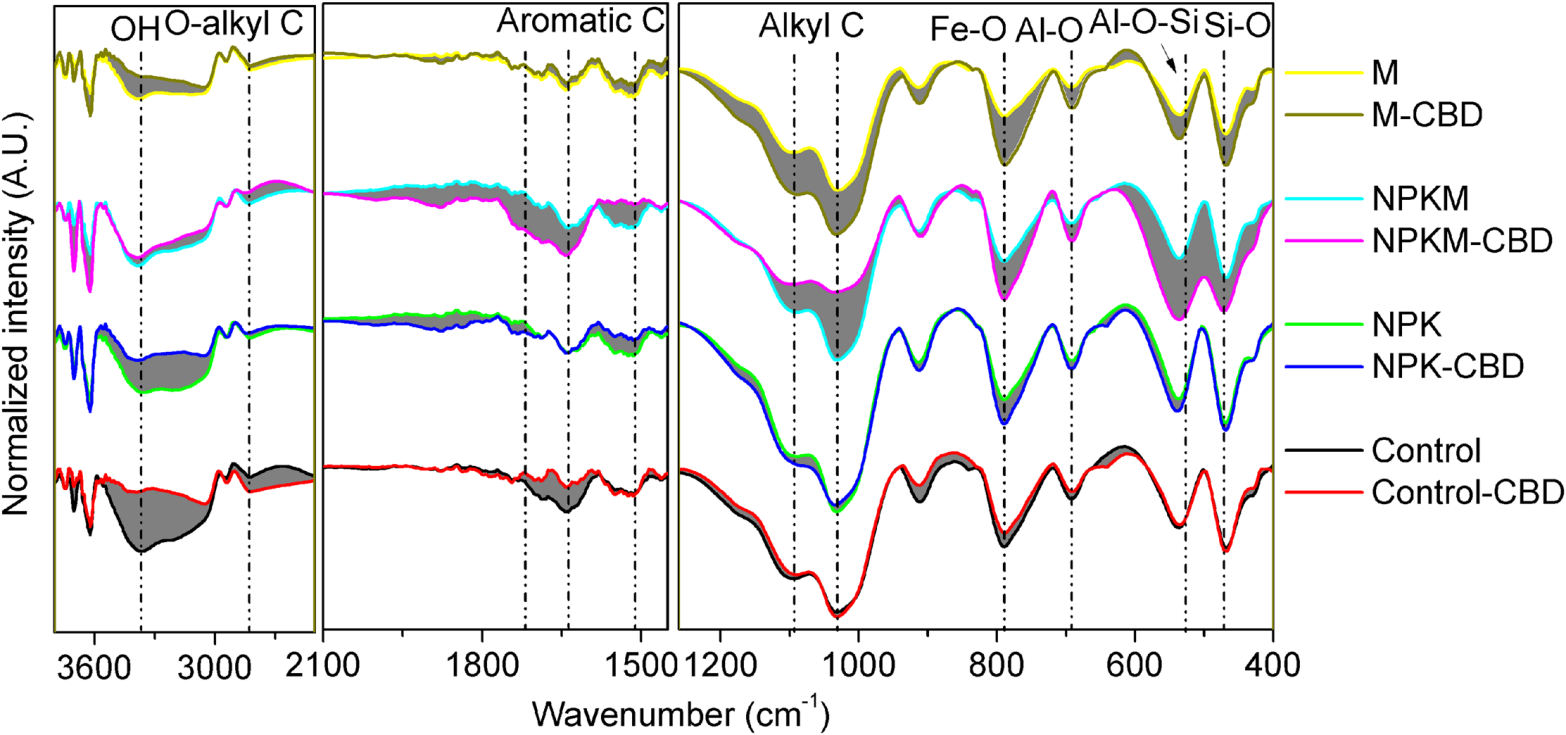
Fourier-transform IR spectra of the CBD extracts and the bulk soils from the variouslong-term fertilisation treatments. Control, no fertilisation; NPK, chemical fertilisation; NPKM, chemical plus swine manure fertilisation; M, swine manure fertilisation. CBD, citrate-bicarbonate-dithionite. The grey region indicates the extracted reactive minerals and organic C pool, suggesting that considerably greater amounts of reactive mineral components (Fe-O, Al-O, and Si-O) and organic C were extracted from the long-term organic treatments (i.e., M and NPKM) using the CBD method than were extracted from the Control and NPK treatments.

FTIR spectroscopy was used to examine the difference of organic C and minerals in the CBD extracts and bulk soils (Fig 2). For the Control and NPK treatments, the band intensity between the CBD extracts and bulk soils was similar, except for 3465 cm^-1^ (O-H network). Interestingly, for the M and NPKM treatments, the band intensity of the CBD extracts was more stronger at 1640 cm^-1^ (aromatic C), 1100 cm^-1^ (alkyl C), 1020 cm^-1^ (alkyl C), 790 cm^-1^ (Fe-O), 690 cm^-1^ (Al-O), 520 cm^-1^ (Al-O-Si) and 480 cm^-1^ (Si-O) than that of bulk soils. Therefore, FTIR of the CBD extracts and bulk soils confirmed that substantially greater amounts of reactive mineral components (i.e., Fe-O, Al-O, and Si-O) and organic C were extracted from the soils following the M and NPKM treatments using the CBD method than from the Control and NPK treatments (Fig 2). When the CBD method and the FTIR spectra were combined, it was concluded that the Fe_d_/Fe_t_ ratio, rather than Fe_d_, provides a suitable index for the characterisation of the amount of reactive Fe that is related to the preservation of SOC.

### 3.3 Composition of reactive minerals as studied by Fe K-edge XANES spectra

**Fig 3:**
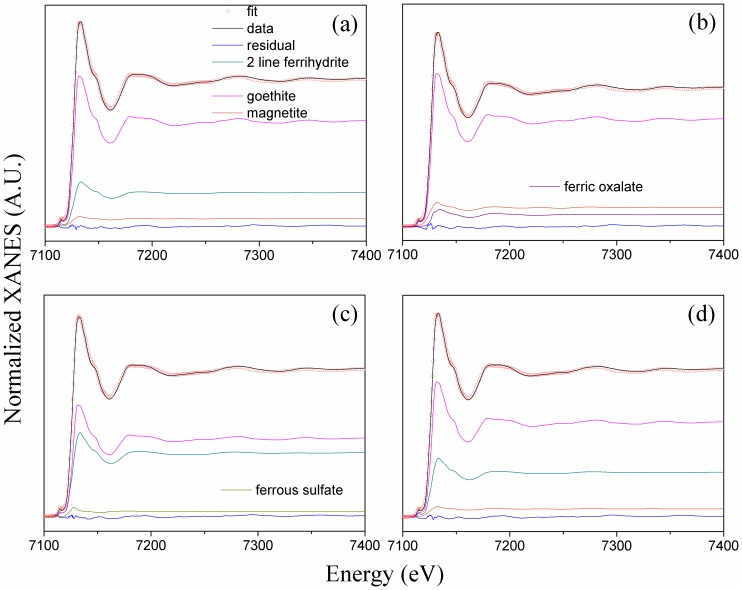
Fe K-edge XANES spectra of soil colloids from contrasting fertilisation treatments. (a) Control, no fertilisation; (b) NPK, chemical fertilisation; (c) NPKM, chemical plus swine manure fertilisation; (d) M, swine manure fertilisation. The scattered circles represent the linear combination fitting (LCF) results of the sample spectra.

To determine the composition of the reactive Fe minerals, the Fe K-edge XANES spectra with the linear combination fitting (LCF) were analysed using eight reference materials (Fig 3). The eight reference materials were ferrihydrite, goethite, lepidocrocite, maghemite, Fe(III) sulphate, Fe(II) sulphate, Fe(III) oxalate, and Fe(II) oxalate (S1 Fig). Of these, ferrihydrite, goethite, lepidocrocite, and magnetite represent the main reactive minerals; Fe(III) sulphate and Fe(II) sulphate represent the primary forms of tri- and bi-valent inorganic irons, while Fe(III) oxalate and Fe(II) oxalate represent the primary forms of tri- and bi-valent organic irons. The LCF results for the soil colloids (Fig 3 and Table 2) revealed that Fe(III) was predominant (71%-84.2%) following both the organic and the chemical fertilisation treatments. The other Fe phases were composed of less crystalline ferrihydrite (15.8%-25.9%) in the organic (i.e., NPKM and M) treatments and more crystalline maghemite (29.0 ± 1.5%) in the chemical (i.e., NPK) treatments. For the Control treatment, the reactive Fe minerals in the soil colloids were composed of 47.5% inorganic Fe(III) and 52.5 ± 2.5% ferrihydrite. Given the greater C preservation capability of ferrihydrite than maghemite [25–27], it is reasonable to conclude that the Fe minerals present in soil colloids after long-term organic fertilisation are more reactive than those present after long-term chemical fertilisation.

**Table 2:** Linear combination fit (LCF) results of the Fe K-edge XANES spectra of soil colloids from various fertilisation treatments and the simulated studies ^a^.

Samples		LCF results (%)					LCF parameters	
		Ferrihydrite	Goethite	Magnetite	Ferrous sulfate	Ferric oxalate	R-factor	Chi-square
fertilisation treatment	Control	23.0 ± 2.3	71.7 ± 1.2	5.3 ± 1.4	ND	ND	0.000042	3.71 × 10^−5^
	NPK	ND	77.6 ± 1.6	13.8 ± 0.7	ND	8.7 ± 0.7	0.000061	5.34 × 10^−5^
	NPKM	43.4 ± 1.4	53.2 ± 1.1	ND	3.4 ± 0	ND	0.000054	4.68 × 10^−5^
	M	30.3 ± 2.5	64.3 ± 1.2	5.4 ± 1.6	ND	ND	0.000048	4.09 × 10^−5^
Simulated studies	M+10 mg/L oxalic acid	53.1±0.013	47.8±1.4	ND	ND	11.3±0.9	0.000713	1.08×10^−4^
	M+100 mg/L oxalic acid	49.7±3.1	40.9±2.5	ND	ND	14.4±0.6	0.000644	1.23×10^−4^

^*a*^Control, no fertilisation;

NPK, chemical fertilisation; NPKM, chemical plus swine manure fertilisation; M, swine manure fertilisation. ND, not detected. The values of the fitting parameters (i.e., R-factor and chi-square) indicate that the fitting results are satisfactory.

### 3.4 Simulation of reactive mineral formation by adding organic acid

The factors that affect the formation of reactive minerals in the natural system were complicated [27]. To confirm the role of organic inputs in the formation process, a simulated study, i.e., adding oxalic acid to soil colloids from the M treatment, was conducted.

**Fig 4:**
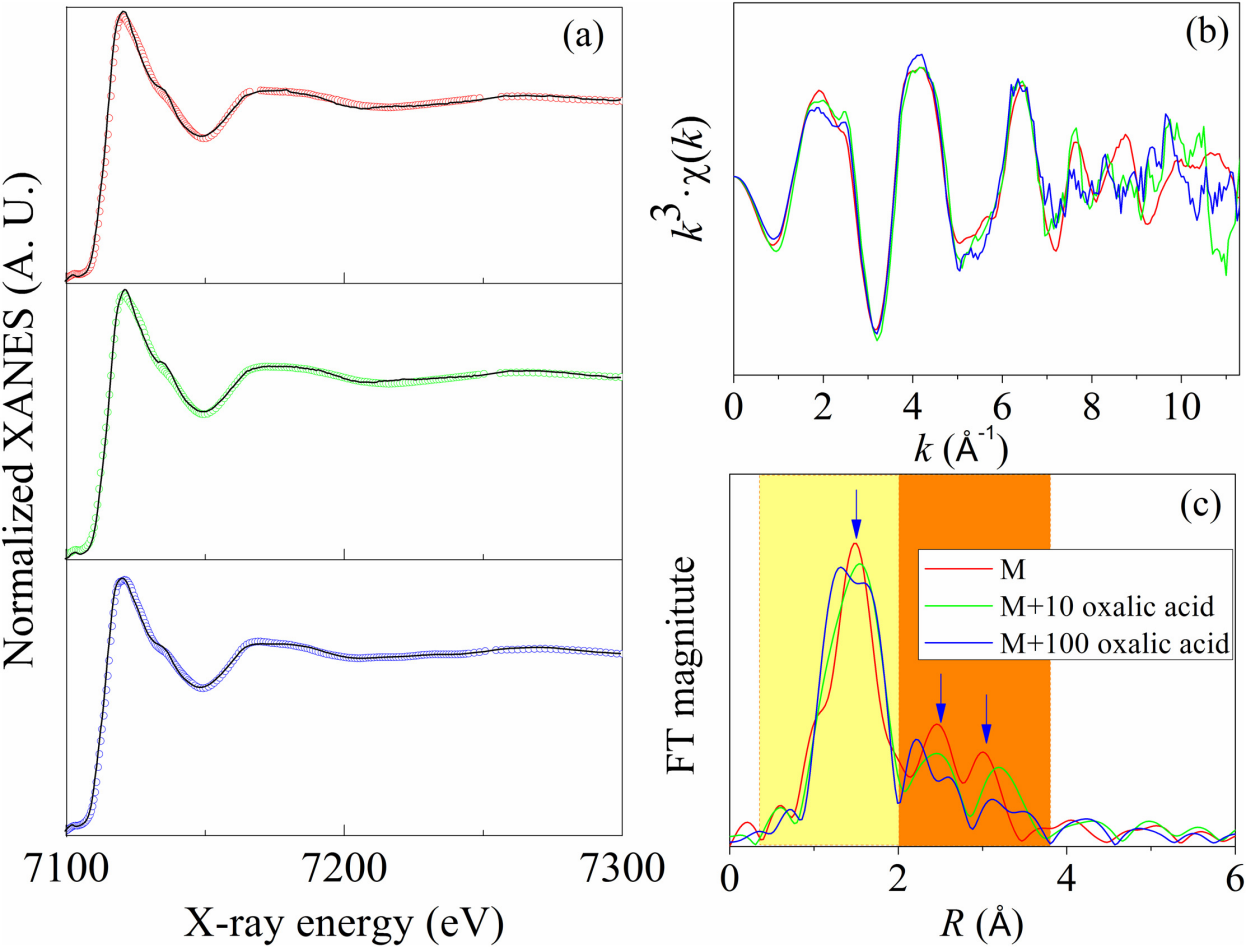
Linear combination fitting (LCF) results of XANES Fe K-edge normalized spectra (a), Fe K-edge EXAFS (b), and radial structure function (RSFs, uncorrected for phase shift) (c) in simulated studies. The scattered circles represent the LCF results of the sample spectra. Fitting parameters (i.e., R-factor and chi-square, Table 2) indicated that the fitting results are convincible.

The LCF results of Fe K-edge XANES spectra (Fig 4-a and Table 2) demonstrated that incubation of soil colloids with oxalic acid at a concentration of 10 and 100 mg/L for 1 day could decrease the percentage of goethite from 64.3 ± 1.2% to 47.8 ± 1.4% and 40.9 ± 2.5%, respectively, and simultaneously increase the percentage of ferrihydrite from 30.3 ± 2.5% to 53.1 ± 0.0% and 49.7 ± 3.1%, respectively. Furthermore, the extended X-ray absorption fine structure (EXAFS) spectra and the radial structure functions (RSFs, uncorrected for phase shift) of those data (Fig 4-b and 4-c) demonstrated that the addition of oxalic acid caused structural changes to Fe minerals, indicative of the occurrence of both co-precipitation and absorption [28] with the addition of oxalic acid to soil colloids in the M treatment.

The above results suggest that oxalic acid can promote the transformation from Fe(III) to ferrihydrite, which is consistent with aprevious report that the low-molecular-weight (LMW) organic acid may incorporate into the network structure of SRO minerals [29], inhibiting further growth of SRO minerals [3].

Ferrihydrite was indicative of recent Fe weathering and forms by the rapid oxidation of Fe(II) in solution [30]. Therefore, its weathering was an important step in the transformation process [27]. Considering the large amounts of Fe in the organic treatment (Table 1), it is suggested that Fe in bulk soils might be reduced to Fe(II) first, and then oxidized to Fe(III) with the assistance of oxidizing substances, and lastly, the Fe(III) is incorporated into the network structure of organic acids to form ferrihydrite.

### 3.5 Organic groups preserved by reactive minerals

**Fig 5:**
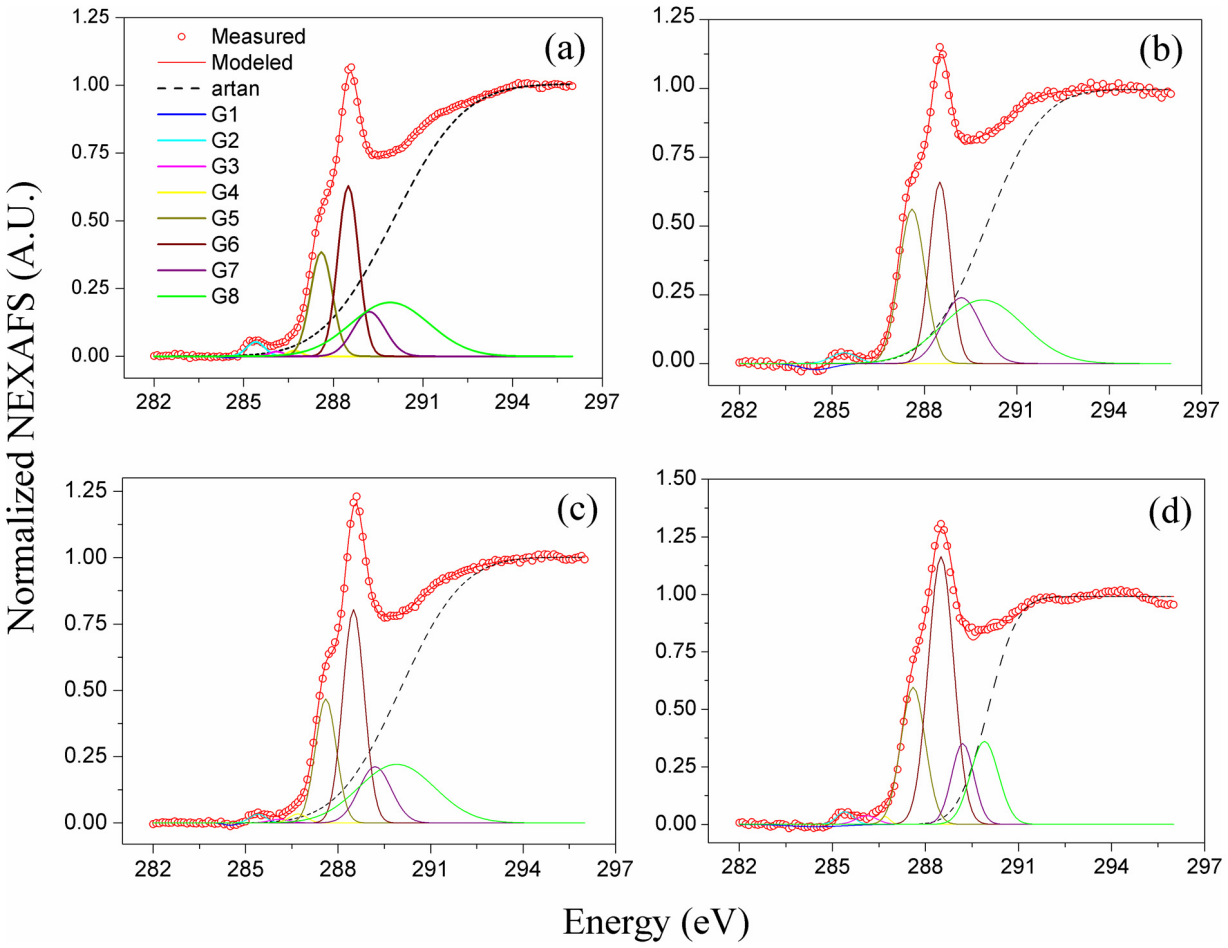
C 1s NEXAFS spectra and their deconvolution results for soil colloids from the various long-term fertilisation treatments. (a) Control, no fertilisation; (b) NPK, chemical fertilisation; (c) NPKM, chemical plus swine manure fertilisation; (d) M, swine manure fertilisation. G1–G8 representeight Gaussian curves. The specific C forms of G1–G8 are given in S1 Table. Artan represents an arctangent step function.

To date, it remains unclear which components of soil organic C are preserved by reactive Fe minerals. Synchrotron-based C 1s near-edge X-ray fine structure (NEXAFS) spectroscopy can offer valuable insights into the composition of organic C [31]. Using C 1s NEXAFS spectroscopy combined with deconvolution analysis [20], it was found that compared to the NPK treatment, the NPKM and M treatments markedly increased the proportion of carboxylic groups (288.4–289.1 eV) from 24.2% to 33.2% and increased the percentages of both the aromatic (283.0–286.1 eV) and phenolic (286.2–287.5 eV) groups by greater than 2.8-fold (Fig 5 and Table 3). These results demonstrated that organic fertilisation treatments enhanced the retention of carboxylic and aromatic C in soils.

**Table 3:** Deconvolution results for using C 1s NEXAFS on soil colloids from the various long-term fertilisation treatments^a^.

Treatment	Proportion of absorption regions (%)					
	Aromatic C	Phenolic C	Alkyl C	Carboxylic C	O-alkyl C	Carbonyl C
	(283–286.1 eV)	(286.2–287.5 eV)	(287.6–288.3 eV)	(288.4–289.1 eV)	(289.2–289.8 eV)	(289.9–290.2 eV)
Control	2.6	0.7	19.4	29.6	12.1	35.6
NPK	0.5	0.1	25.7	24.2	16.3	33.2
NPKM	1.4	1.1	18.9	33.2	13.2	32.2
M	1.8	0.5	22.9	46.6	12.3	15.9

^*a*^Control, no fertilisation;

NPK, chemical fertilisation; NPKM, chemical plus swine manure fertilisation; M, swine manure fertilisation.

To determine whether the retention of C is directly correlated with the content of reactive Fe minerals, the Pearson correlations between C functional groups and Fe_t_ as well as Fe_d_ were evaluated (Table 4). The results showed that total Fe was significantly correlated with the carboxylic C groups (*P* < 0.001, *R*^2^ > 0.69) but was not significantly correlated with the other C groups. However, the CBD extracted Fe was significantly correlated with aromatic C and O-alkyl C functional groups (*P* < 0.001, *R*^2^ > 0.77). Therefore, it is reasonable to conclude that reactive Fe minerals are responsible for the retention of aromatic C and O-alkyl C in soils.

**Table 4:** Pearson correlation coefficients amonghighly reactive Fe fractions (i.e., Fe_d_) and C functional groups (n = 12)^a^.

Functional group	Energy level (eV)	Fe_d_		Fe_t_	
		*P*	*R*^2^	*P*	*R*^2^
Aromatic C	283.0–286.1	<0.001	0.77	0.062	0.23
Phenolic C	286.2–287.5	<0.05	0.28	0.065	0.23
Alkyl C	287.6–288.3	<0.05	0.40	0.111	0.15
Carboxylic C	288.4–289.1	<0.01	0.48	<0.001	0.69
O-alkyl C	289.2–289.8	<0.00001	0.99	<0.01	0.61
Carbonyl C	289.9–290.2	0.16	0.10	<0.05	0.34

^*a*^Fe_d_, the citrate-bicarbonate-dithionite extracted Fe. Fe_t_, the total Fe.

## Discussion

It is well known that, compared to chemical fertilisation treatments, long-term organic fertilisation treatments can increase soil C pools [8,9,32]. However, the effects of long-term organic fertilisation treatments on the release of mineral elements are poorly understood. The release of mineral elements in soils was considered a precursor to the formation of reactive minerals [33]. The results in this study demonstrated that organic fertilisation treatments resulted in the release of considerable amounts of mineral elements into soil colloids, which were significantly (*P* < 0.01) correlated with the concentration of DOC. This result was also consistent with the higher degree of soil weathering that received organic fertilisation treatments (Table 1). It had been suggested that the weathering of soil was driven by microorganisms [34], the diversity and function of which were affected by nutrient availability [35]. Here, it is inferred that the organic fertilisation treatments not only provided a high availability of nutrients (i.e., manure) to microorganisms but also created a suitable environment (e.g., correct pH) for microbial activity. Thus, on the one hand, microbes were present in high abundance, which promotes the weathering of soil and on the other hand, more rapid weathering provided a greater availability of nutrients for the formation of reactive minerals.

Analysis of the concentrations of ferrihydrite in the applied fertilisers and the XRF spectroscopy results [36] indicated that ferrihydrite introduced by fertilisers was negligible, supporting that fertilisers had a minor effect on the concentration of reactive minerals in soil. Furthermore, the results of simulated studies demonstrated that the addition of organic acid could transfer goethite to ferrihydrite (Fig 4), providing direct evidence that organic inputs promoted the formation of reactive minerals. The presence of organic matter was critical to the formation of reactive minerals because organic matter could become incorporated into the network structure of short-range ordered (i.e., SRO) minerals and thus prevent the formation of sheets or inter-layer H-bonds that were essential components of crystalline minerals [8,37,38]. Moreover, high concentrations of organic matter might promote the formation of reactive Fe minerals by inhibiting further growth of reactive Fe minerals to their crystalline counterpart [3].

Therefore, soils that receive long-term swine manure inputs contained high concentrations of organic matters (mainly as carboxylic C, alkyl C, carbonyl C, and O-alkyl C, Table 3) that facilitated the rapid formation of reactive Fe minerals. The formation of reactive Fe minerals facilitated the storage of SOM, which was perhaps the most important determinant of soil quality and soil sustainability [39]. It had been shown that the quality of the land and so its suitability in China had decreased, based on the second National Land Resource Survey of China [40]. Therefore, it is urgent for China to improve the quality of low- and medium-grade arable land. One of the effective ways to improve the quality of the land and its suitability of the land was adding manure to soil [41]. Moreover, the comparison between the FTIR spectra of the CBD extracts and those of the bulk soils has demonstrated, for the first time, that FTIR spectroscopy is a suitable method for the characterisation of reactive Fe minerals related to the preservation of SOC.

Reactive Fe minerals had been suggested to play an important role in preserving soil C. However, it is unclear as to which Fe minerals are responsible for the preservation of soil C. Therefore, the identification of reactive Fe minerals is critical for improving our understanding of the mechanisms underlying the preservation of soil C by reactive Fe minerals. The identification of reactive Fe minerals is still a complicated issue, owing to the limitations of traditional techniques (i.e., X-ray diffraction) that had been used to identify SRO minerals as the main components of reactive Fe minerals [42]. Fe K-edge XANES spectroscopy clearly demonstrated that organic fertilisation treatments promoted the formation of reactive Fe minerals, i.e., ferrihydrite, compared to chemical fertilisation treatments. These results suggested that even when the quantities of Fe minerals were similar, the forms of Fe minerals present in organic fertilised soils played a more important role in the preservation of soil C than those present in inorganic fertilised soils. A better understanding of the effects of fertilisation practices on reactive iron oxides is important for predicting and managing C preservation in soils.

## Conclusion

In summary, these findings demonstrated that long-term organic fertilizer treatment increased the mobilization of Fe minerals. Moreover, the interaction between organic materials and Fe minerals may facilitate the rapid formation of reactive Fe minerals in soil colloids. These findings raise new possibilities for investigating the regulation of reactive soil minerals and their roles in efficient C preservation, in addition to providing a pathway for predicting and managing the global C cycle.

## Supporting Information

S1 Fig Fe K-edge XANES spectra of reference materials. (PDF)

S1 Table Peak assignment for C forms obtained from C 1s NEXAFS and corresponding FTIR spectroscopy [12,17,40]^*a*^. (PDF)
