# Supplementary figures and images for: Spectroscopic Evidence of the Improvement of Reactive Iron Mineral Content in Red Soil by Long-Term Application of Swine Manure
Source: PLoS One. 2016 Jan 11;11(1):e0146364. doi: 10.1371/journal.pone.0146364 (PMC4713869; doi:10.1371/journal.pone.0146364)

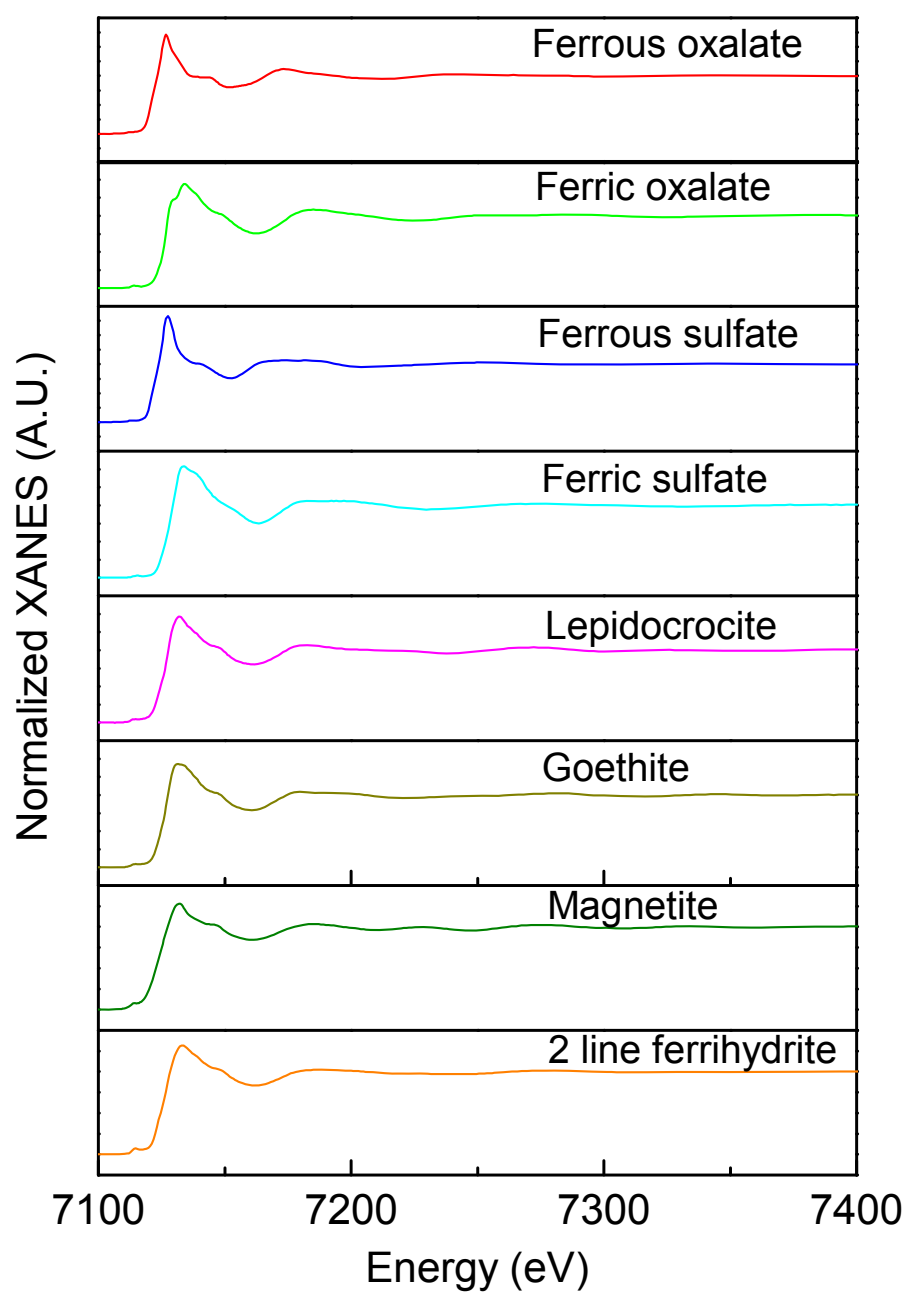

18

19 **S2 Fig.** Fe K-edge XANES spectra of reference materials.

Supplement: S1 Fig — (PDF) [file pone.0146364.s001.pdf]
