# Supplementary material for: Spectroscopic Evidence of the Improvement of Reactive Iron Mineral Content in Red Soil by Long-Term Application of Swine Manure
Source: PLoS One. 2016 Jan 11;11(1):e0146364. doi: 10.1371/journal.pone.0146364 (PMC4713869; doi:10.1371/journal.pone.0146364)
Supplement: S1 Table — (PDF) [file pone.0146364.s002.pdf]

Table S1 Peak assignment for C forms obtained from C 1s NEXAFS and corresponding FTIR spectroscopy [a]

Deconvolution curve	Carbon forms	Energy level (eV)	Fit position	Functional group	FTIR Spectroscopy (cm^{-1})
G1	Aromatic C, Quinone-C: C=O	283.0-284.5	284.5		
G2	Carbonyl-substituted Aromatic C: C=C	284.9-285.5	285.4	Aromatic type C	1600-1650
G3	Unsaturated C: C=O	285.8-286.2	286.1		
G4	Phenol-C, ketone: C=O; R-(C=O)R'; C=C-OH	286.0-287.4	286.7	Aromatic C with side chain substituent	1513-1515
G5	Alkyl C: C-H	287.0-287.6	287.6	Alkyl C	1030-1080
G6	Aliphatic-C, aromatic carbonyl	288.0-288.7	288.5	Carboxylic C	1720
G7	Carbonyl-C, alcohol O-alkyl C: C-OH	289.2-289.5	289.2	O-alkyl C	2850-2920
G8	O-alkyl C/carbonyl	289.5-290.2	289.9		

[a] The deconvolution parameters were referred from Heymann et al. (2011)[17] and Schumacher et al. (2005)[12]; the FTIR parameters were referred from Hodgkins et al. (2014)[40].
